# Profile of MIF in Developing Hippocampus: Association With Cell Proliferation and Neurite Outgrowth

**DOI:** 10.3389/fnmol.2020.00147

**Published:** 2020-08-12

**Authors:** Xuejun Chai, Wei Zhang, Lingling Li, Yongji Wu, Xiaoyan Zhu, Shanting Zhao

**Affiliations:** ^1^College of Basic Medicine, Xi’an Medical University, Xi’an, China; ^2^College of Veterinary Medicine, Northwest A&F University, Yangling, China

**Keywords:** MIF, hippocampal development, interneurons, neurite outgrowth, cell proliferation

## Abstract

Proinflammatory cytokine macrophage migration inhibitory factor (MIF) is a multifunctional cytokine and has been found involved in many neurological diseases such as Alzheimer disease (AD), epilepsy, and multiple sclerosis. Previous studies have shown that MIF is expressed in neocortex, hippocampus, hypothalamus, cerebellum, and spinal cord in adult mice. It is expressed by astrocytes and activates microglias in neuroinflammation. Further studies have shown that MIF is detected in moss fibers of dentate granule cells and in apical dendrites of pyramidal neurons in adult hippocampus. Only NeuroD-positive immature granule neurons but not NeuN-positive mature neurons express MIF. These findings led us eager to know the exact role of MIF in the development of hippocampus. Therefore, we systematically checked the spatial and temporal expression pattern of MIF and characterized MIF-positive cells in hippocampus from mice aged from postnatal day 0 (P0) to 3 months. Our results showed that the lowest level of MIF protein occurred at P7 and *mif* mRNA increased from P0, reached a peak at P7, and stably expressed until P30 before declining dramatically at 3 months. MIF was localized in fibers of GFAP- and BLBP-positive radial glial precursor cells in dentate gyrus (DG). DCX-expressing newly generated neurons were MIF-negative. Inhibition of MIF by MIF antagonist S, R-3-(4-hydroxyphenyl)-4, 5-dihydro-5-isoxazole acetic acid methyl ester (ISO-1) reduced BrdU-positive cells. Interestingly, MIF was expressed by NeuN-positive GABAergic interneurons including parvalbumin-and Reelin-expressing cells in the DG. Neither NeuN-positive granule cells nor NeuN-positive pyramidal neurons expressed MIF. In transgenic mice, POMC-EGFP–positive immature dentate granule cells and Thy1-EGFP–positive mature granule cells were MIF-negative. Treatment of neuronal cultures with ISO-1 inhibited neurite outgrowth. Therefore, we conclude that MIF might be important for feature maintenance of neural stem cells and neurite outgrowth during hippocampal development.

## Introduction

Macrophage migration inhibitory factor (MIF) is a proinflammatory cytokine with molecular weight of 12 kDa and is released into the circulation by the anterior pituitary gland as a consequence of central nervous system (CNS) injury or as toxic response to endotoxemia (Bernhagen et al., 1993). It accumulates in neoplastic astrocytes and promotes microglial activation in neuroinflammation (Bacher et al., 2002; Cox et al., 2013). MIF has been found to be expressed in neocortex, hippocampus, hypothalamus, cerebellum, choroid plexus, and spinal cord (Nishibori et al., 1997; Bacher et al., 1998; Ogata et al., 1998; Vedder et al., 2000; Li et al., 2008; Danış et al., 2011; Zhang et al., 2019) and involved in Parkinson disease, Alzheimer disease (AD), autism spectrum disorders, multiple sclerosis, schizophrenia, and gliomas (Grigorenko et al., 2008; Mittelbronn et al., 2011; Nicoletti et al., 2011; Cox et al., 2013; Zeiner et al., 2015; Kassaar et al., 2017; Okazaki et al., 2018; Cheng et al., 2020). In AD patients, the concentration of MIF remarkably increased in the cerebrospinal fluid (Bacher et al., 2010; Zhang et al., 2019). Studies have demonstrated that dysfunction of MIF in AD is due to glycation and oxidation of MIF, which inhibit MIF activity to stimulate glial cells leading to deficiency of clearance of amyloid β (Aβ) protein (Kassaar et al., 2017). The binding assay revealed that MIF expression largely associates with Aβ deposits in AD brain (Zhang et al., 2019). In human gliomas, MIF is strongly expressed by tumor cells and its receptors CD74 is only restricted to microglial cells (Zeiner et al., 2015).

In addition to the role in neuropathological diseases, MIF has been reported to be involved in neurogenesis of hippocampus in normal conditions. MIF colocalizes with vimentin and GFAP in hippocampal precursor cells and astrocytes. Loss of MIF leads to reduced expression of PSA-NCAM and DCX in hippocampus (Conboy et al., 2011; Djordjevic et al., 2017).

Previous studies have indicated that MIF is expressed by astrocytes and locates in mossy fibers of dentate granule cells in adult hippocampus (Bacher et al., 1998; Ogata et al., 1998; Bacher et al., 2002; Cox et al., 2013). Few studies have successfully detected MIF immunostaining in neuronal somata, although *in situ* hybridization shows a strong labeling of *mif* mRNA in cytoplasm of granule cells and pyramidal neurons of hippocampus (Bacher et al., 1998). Until 2011, Conboy and his colleagues demonstrated that MIF is expressed by NeuroD-positive immature granule neurons in hippocampus, but not by NeuN-positive mature neurons (Conboy et al., 2011). However, it is still elusive on MIF expression and characteristics, as well as potential functions, especially in the stage of hippocampal development. To address this issue, we systematically examined the spatial and temporal expression pattern of MIF protein and *mif* mRNA by Western blotting and real-time polymerase chain reaction (PCR) and characterized MIF-positive cells in hippocampus of mice aged from postnatal day 0 (P0) to 3 months by immunostaining. Using antagonist of MIF, we blocked MIF function and observed impairment of cell proliferation and neurite outgrowth in hippocampus. Our results suggest that MIF might maintain the feature of neural stem cells and promote outgrowing of neuronal processes in developing hippocampus.

## Materials and Methods

### Animals

P7 POMC-EGFP and 3-month Thy1-EGFP transgenic mice were obtained from the animal facility of Hamburg University, UKE, Germany. Animals were housed under standard laboratory conditions in the animal facility at the Center for Molecular Neurobiology Hamburg. All experiments were performed in accordance with the institutional guide for animal care (license number ORG 850). Genotyping was performed by PCR analysis of genomic DNA, as described previously (Overstreet et al., 2004; Vuksic et al., 2008). P0, P3, P7, P14, P30, and 3-month Kunming mice were obtained from the Experimental Animal Center of Xi’an Jiaotong University. All procedures were performed in accordance with the protocol approved by the Institutional Animal Care of Northwest A&F University and Xi’an Medical University.

### Antibodies and Inhibitor

The following primary antibodies were used for immunofluorescence studies and Western blot analyses: rabbit polyclonal anti-MIF (1:500, ABclonal, United States), mouse monoclonal anti-Reelin G10 (dilution 1:1,000, Millipore, United States), mouse monoclonal anti-Parvalbumin (1:500, Abcam, United States), mouse polyclonal anti-GFAP (1:500, DAKO, United States), mouse monoclonal anti-NeuN (1:500, Millipore, United States), mouse monoclonal anti–neurofilament 200 (1:1,000, Millipore, United States), mouse monoclonal anti–β-tubulin (1:500; Sigma-Aldrich, United States), goat polyclonal anti-DCX (1:1,000; Santa Cruz, United States), mouse monoclonal anti-BLBP (1:300, Raybiotech, Inc., United States), and rat polyclonal anti-BrdU (1:500, Bio-Rad Laboratories Inc., United States). The secondary antibodies used for Western blotting were horseradish peroxidase (HRP)–conjugated goat anti–rabbit IgG and goat anti–mouse IgG (1:2,000; Cell Signaling Technology, United States). The secondary antibodies for immunostaining were as follows: Alexa Fluor 488-conjugated goat anti–mouse, Alexa Fluor 568–conjugated goat anti–mouse, Alexa Fluor 488-conjugated goat anti–rabbit IgG, Alexa Fluor 568–conjugated goat anti–rabbit IgG, Alexa Fluor 488-conjugated donkey anti–goat, Alexa Fluor 488–conjugated goat anti-rat. Dyes used were DAPI (1:10,000; Invitrogen, United States) and TRITC-labeled phalloidin (1:4,000; Sigma, United States).

S, R-3-(4- hydroxyphenyl)-4, 5-dihydro-5-isoxazole acetic acid methyl ester (ISO-1), an antagonist of MIF, was purchased from Calbiochem, Germany. BrdU was purchased from Sigma-Aldrich, United States.

### Plasmids Construction

The procedures of pCAG-EGFP and pCAG-MIF-MYC construction were identical as previously described (Zhang et al., 2014). In brief, the MYC tag was inserted into the vector pCAG-MCS (Biowit Technologies, China) by using In-Fusion^®^ HD Cloning Kit (Clontech, United States) according to the manufacturer’s protocol, and then the full-length MIF was cloned into pCAG-MCS-MYC by the traditional cloning method. pCAG-EGFP was obtained by the insertion of EGFP fragment into linearized pCAG-MCS using the traditional cloning method.

### HEK293T Cell Culture and Transfection

HEK293T cells were grown in DMEM (Dulbecco modified eagle medium, low glucose; Invitrogen) with 10% fetal calf serum (FCS; Invitrogen) and 1% penicillin-streptomycin (P/S, 10,000 U/mL; Invitrogen) at 37°C in a humidified atmosphere of 5% CO_2_. When reaching 60–70% confluence in 60 mm culture dishes, HEK293T cells were transfected with pCAG-MIF-MYC using Lipofectamine 2000 Kit (Thermo Fisher Scientific, United States). Forty-eight hours after transfection, the cells were collected, frozen in liquid nitrogen, and stored at -80°C for Western blotting.

### *In utero* Electroporation and Overexpression of MIF in Migrating Neurons

*In utero* electroporation was performed according to the procedure described as previously (Chai et al., 2015, 2016). Timed-pregnant females were deeply anesthetized by intraperitoneal injection of chloral hydrate (4.3 mg per 10 g of body weight). Then, the uterine horns of the pregnant mice were carefully exposed via a midline abdominal incision by pinching gaps between embryos with ring-forceps. Approximately 1–1.5 μL of pCAG-MIF-MYC and pCAG-EGFP (1 mg/mL for each) plasmid solution was injected into the lateral ventricle of mouse embryos on embryonic day 15.5 (E15.5) using a mouth-controlled pipette system and a pulled-glass micropipette. Fast green solution (0.1%) was added to the plasmid solution in a ratio of 1:10 to monitor the injection. The heads of injected embryos in the uterus were placed between the tweezers-type electrodes. Electronic pulses (30–50 V; 50 ms) were charged five times at intervals of 900 ms with an electroporator (Gene Pulser Xcell Electroporation System, United States). The uterine horns were placed back into the abdominal cavity to allow the embryos to continue normal development until sacrificed at P0. Brains (*n* = 9) were collected and fixed with 4% paraformaldehyde (PFA) at 4°C overnight. After rinsing in 0.1 M PB, brains were embedded in 5% agar and cut into 40-μm-thick slices on a Leica VT 1000S vibratome (Leica Microsystems, Germany). The sections were placed in a 24-well plate containing 0.1 M PB for immunostaining.

### Preparation of Protein Extracts

P0 and P3 Kunming mice were sacrificed under frozen anesthesia. P7, P14, P30, and 3-month Kunming mice were anesthetized with an overdose of Narkodorm (500 μL/kg) first with 0.9% NaCl and decapitated (*n* = 4 for each). Hippocampi were prepared and frozen with liquid nitrogen for immediate protein extraction or stored at -80°C. Tissue pellets and cell cultures were taken out from -80°C and incubated in ice-cold hypertonic lysis buffer [pH 7.6, 50 mM Tris-HCl, 150 mM NaCl, 5 mM EDTA-Na_2_, 1% (vol/vol) Non-idet P-40, 1% Triton X-100, 0.5% (wt/vol) sodium dodecyl sulfate (SDS), 0.25% (wt/vol) sodium deoxycholate] with 1% protease inhibitor (Sigma, United States), and phosphatase inhibitor cocktails (Sigma). The tissue pellets and cells were lysed by repeated freezing in liquid nitrogen and thawing at 37°C (five times). After the tissue was triturated with a pipette tip to homogenize larger pieces of tissue and sonicated for 5 min, the suspension was centrifuged at 20,000 × *g* at 4°C for 30 min. The resulting crude supernatants were taken, and protein concentration was measured by using BCA Protein Assay Kit (Pierce, United States). Aliquots of the supernatants were immediately used for Western blotting or were stored at -80°C until use.

### Western Blot Analysis

Five to 10 μg of each sample was diluted in ultrapure water containing 1 × NuPAGE sample buffer (Invitrogen) and 1 × NuPAGE reducing agent (Invitrogen) to final volumes of 24 μL, boiled at 95°C for 5 min, and then immediately placed on ice. The samples were separated by NuPAGE bis-tris, pH 7.0, 4–12% PAGE (Invitrogen) with 1 × NuPAGE MES-SDS running buffer (Invitrogen), and transferred electrophoretically to polyvinylidene fluoride membranes with 1 × NuPAGE transfer buffer (Invitrogen). For blotting, membranes were blocked with 5% (wt/vol) non-fat milk or 5% bovine serum albumin (BSA) in tris-buffered saline at room temperature (RT) for 1 h and finally incubated with primary antibodies in blocking solution at 4°C overnight. After three washes, the blots were subsequently incubated with a HRP-conjugated goat anti–rabbit or mouse IgG secondary antibody (1:2,000; Cell Signaling, United States) for 2 h at RT. The immunoreaction was visualized by enhanced chemiluminescence and scanned with a chemiluminescent and fluorescent imaging system (BioRad, United States). Bands were normalized to β-tubulin level to provide a control for equal loading. At least three Western blots were analyzed for each experiment. Blots were quantified by densitometry using ImageJ (NIH, United States). Significance was determined by one-way analysis of variance across all samples followed by Tukey test (^∗^*P* < 0.05, ^∗∗^*P* < 0.01, ^∗∗∗^*P* < 0.001) (GraphPad Prism 5, United States).

### Quantitative Real-Time PCR

Quantitative real-time PCR was performed according to the procedure described as previously (Zhang et al., 2014). In brief, P0, P3, P7, P14, P30, and 3-month Kunming mice (*n* = 4 for each) were anesthetized, and decapitated; hippocampi were prepared and frozen with liquid nitrogen for immediate total RNA extraction or stored at -80°C. Total RNA was extracted using Trizol reagent (Invitrogen, United States). The integrity of RNA was verified by 1% agarose gel, and cDNA was synthesized from 1 μg of total RNA primed with oligo (dT) using RevertAid First Strand cDNA Synthesis Kit (Thermo Scientific, United States). Quantitative PCR was performed using the Bio-Rad CFX96 (Bio-Rad, CA, United States) with Actb as an internal control. All PCR products span an intron in the genomic DNA. The cDNA was amplified with SYBR Premix Ex TaqTM (Takara, Japan). Relative quantification of mRNA levels was performed using the comparative Ct method.

### Neuronal Cultures

Dissociated cultures of hippocampus were prepared as described previously (Chai et al., 2009). For primary cell cultures, newborn (P0) Kunming mice (*n* = 9) were used. The hippocampi were dissected and put into ice-cold Hanks balanced salt solution (HBSS). After removal of the meninges, the hippocampi of both hemispheres were collected and maintained in 15 mL Falcon tubes (BD Biosciences, United States) containing 3 mL of 0.5% Trypsin and 0.53 mM EDTAx4Na (Invitrogen, United States) and incubated at 37°C for 10 min. The hippocampi were then washed twice in ice-cold HBSS and manually triturated with a polished glass Pasteur pipette for several times. Then, the cells were centrifuged at low speed (800 × *g*) for 3 min to discard dead cells. The sediment was collected in fresh Falcon tubes and centrifuged again. The supernatant was discarded, and cell pellets were resuspended in 1 mL of neurobasal-A medium (Invitrogen, United States), supplemented with 2% B27 (Invitrogen, United States), 1 mM Glutamax (Invitrogen, United States), 100 U/mL penicillin, and 100 μg/mL streptomycin (Invitrogen, United States). Hippocampal cells were counted using a hemocytometer and suspended at a density of 5 × 10^5^ cells in 0.5 mL aliquots on glass coverslips inserted in 24-well plates. The coverslips had been coated overnight with a solution of 20 μg/mL poly-L-ornithine (Sigma, United States) prepared in borate buffer, pH 8.1. After 2 h of incubation in a humidified incubator at 37°C and 5% CO_2_ to allow for cell attachment, the medium was changed to remove dead cells. After 12 h of incubation, cultures were divided into three groups (*n* = 12 wells for each group): one group was allowed to grow about 5 days. The second group was used for ISO-1 treatment for 48 h. The third group was used for control treatment for 48 h. Then the cultures were fixed with 4% PFA in 0.1 M PB, pH 7.4, for 15 min at RT and rinsed in 0.1 M PB for 1 h followed by incubation in blocking solution for immunostaining.

### ISO-1 Treatment *in vitro*

ISO-1 (Calbiochem, Germany) was freshly dissolved in dimethyl sulfoxide (DMSO). Twelve hours after seeding of neurons in 24-well dishes, medium was replaced by serum-free medium containing 10 or 100 μM ISO-1, and neurons were incubated for 48 h before fixing with 4% PFA in 0.1 M PB for immunostaining.

### ISO-1 Treatment *in vivo*, BrdU Injection, and Labeling

P5, P14, P30, and 3-month Kunming mice (*n* = 4 for each) were injected with BrdU [intraperitoneal (i.p.), 50 mg/kg; Sigma-Aldrich, United States] dissolved in physiological saline 2 h (evidence proliferating cells only, Conboy et al., 2011) before being anesthetized with an overdose of Narkodorm (500 μL/kg) with 0.9% NaCl and then transcardially perfused with 4% PFA in 0.1 M PB (pH 7.4). For MIF inhibition experiment, P6 Kunming mice (*n* = 8) were injected with ISO-1 (i.p., 7 mg/kg; Calbiochem, Germany) or vehicle (DMSO, i.p.) daily for 9 consecutive days. On P15, mice were injected with BrdU 4 h (to evidence proliferating cells only, due to the blocking effect of MIF by ISO-1, the incubation time was elongated) before being anesthetized and perfused with 4% PFA. Brains were postfixed with 4% PFA in 0.1 M PB overnight at RT, washed 24 h, and cut into 50 μm thick sections on a Leica VT 1000S vibratome (Leica Microsystems, Germany).

BrdU-labeled sections were denatured by incubation with 2 M HCl for 30 min at 37°C and washed with 0.1 M borate buffer (pH 8.0) for several times. After rinsing with 0.1M PB three times, sections were placed in a 24-well plate for immunohistochemistry.

### Immunohistochemistry

P0 mice were decapitated under hypothermic anesthesia; brains were removed and immersion fixed in 4% PFA. P7, P14, P30, and 3-month mice (including POMC-EGFP and Thy1-EGFP transgenic mice) were anesthetized with an overdose of Narkodorm (500 μL/kg) first with 0.9% NaCl and then transcardially perfused with 4% PFA in 0.1 M PB (pH 7.4). Brains were removed and postfixed in 0.1 M PB (PH7.4) containing 4% PFA overnight at RT. After washing in 0.1 M PB overnight, fixed brains were embedded in 5% agar and cut coronally into 40-μm-thick slices to contain the entire hippocampus on a Leica VT 1000S vibratome (Leica Microsystems, Germany). After washing for 2 h at RT in 0.1 M PB, sections were placed in a 24-well plate containing 0.1 M PB for immunostaining. Sections together with sections from *in utero* electroporation, BrdU experiment, and neuronal cultures treated with ISO-1 and control medium were preincubated with blocking solution (5% normal goat serum and 1% BSA in 0.1 M PB containing 0.2% Triton X-100) at RT for 1 h and then incubated with the primary antibodies diluted in blocking solution overnight at 4°C. After washing in 0.1 M PB for 15 min three times, sections or cultured cells were incubated in secondary antibodies diluted in 0.1 M PB for 2 h at RT. After washing in 0.1 M PB for 15 min three times, sections or cultured cells were counterstained with DAPI (1:10,000; Invitrogen). Some cell cultures were stained for F-actin with TRITC-labeled phalloidin (1:4,000; Sigma). After rinsing in 0.1 M PB 15 min three times, sections and cells were mounted with fluorescent mounting medium (Dako North America, Inc., United States) and photographed by a structured-illumination microscope (Zeiss observer Z1) or using a confocal laser scanning microscope (Olympus FV1000).

### Quantitative Assessments

Quantifications of double-labeled cells with MIF and NeuN, MIF and GFAP, MIF and parvalbumin (Pv), and MIF and Reelin were performed using ImageJ software.

Morphometric measurements of cultured hippocampal neurons treated with vehicle, 10 μM ISO-1, and 100 μM ISO-1 were performed by ZEN 2011 software. Data were from three independent neuronal cultures. At least three fields were randomly photographed in each condition of every experiment; 2.5 DIV neurons have differentiated with a longest neurite, which later will develop into axons. Neurites were traced along the processes. The longest neurite, number of terminal tips, and total length of neurites were analyzed by GraphPad Prism 5. One-way analysis of variance with Tukey post test and Kruskal–Wallis test with Dunn post test were performed for parametric and non-parametric data, respectively. Graphs were represented as the mean ± SEM. Differences were considered significant when *P* < 0.05, and represented as ^∗^*P* < 0.05, ^∗∗^*P* < 0.01, ^∗∗∗^*P* < 0.001.

Quantification of newly generated cells was calculated by counting BrdU-positive cells in 1,000 μm sectional length in the SGZ and granular cell layer (GCL) of the hippocampus from P6 mice injected with either DMSO or ISO-1 for consecutive 9 days. Totally, 12 coronal sections for each group were photographed at 20× magnification with a Zeiss microscope. Values were analyzed for significance using the Student two-tailed *t*-test (GraphPad Prism 5).

## Results

### MIF Antibody Is Verified to Be Sufficiently Detectable *in vivo*

**FIGURE 1:**
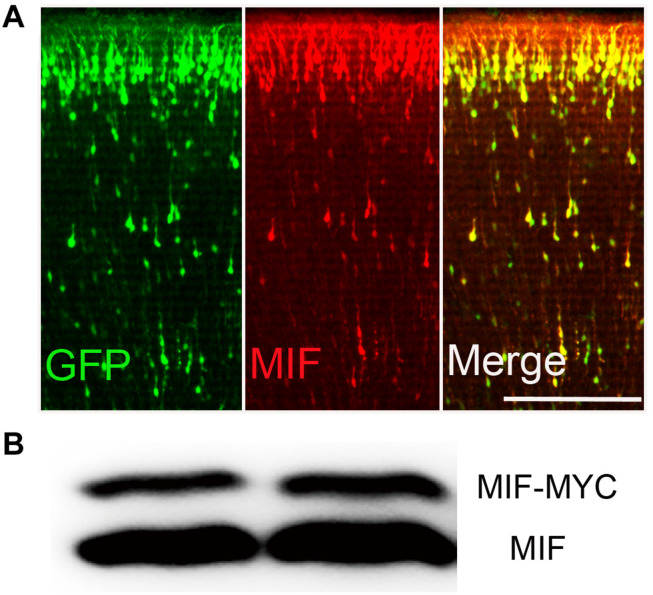
MIF antibody verification. **(A)** Embryonic cortical neurons were cotransfected with pCAG-MIF-MYC and pCAG-EGFP using *in utero* electroporation at E15.5 and fixed at P0. Sections were then immunostained with antibody against MIF (red). Strong labeling of MIF was found in cytoplasm and neurites of GFP-labeled neurons. **(B)** Western blotting showed that MIF antibody recognized endogenous MIF (lower band) and exogenous MIF (MIF-MYC, upper band) expressed by transfected-293 tumor cells. Scale bar in **(A)**: 200 μm.

In order to identify the effectiveness of MIF antibody purchased commercially, plasmid pCAG-MIF-MYC expressing MIF and MYC tag and plasmid pCAG-EGFP expressing EGFP were constructed and electroporated into embryonic cortical neurons. After birth of mice, brains were collected and cut into sections for MIF immunostaining. We found that neurons transfected with EGFP and MIF plasmids on E15.5 have reached their final destinations in the superficial layer of the cortex. They were perfectly recognized by MIF immunostaining (Figure 1A). Plasmid pCAG-MIF-MYC was also transfected into cultured HEK293T cells. Cells were harvested, and MIF protein expression was analyzed by Western blotting. As shown in Figure 1B, MIF antibody detected not only endogenous MIF-MYC, but also exogenous MIF (Figure 1B). Our observations indicate that MIF antibody purchased commercially is specific and sufficient to examine MIF expression by immunostaining.

### MIF Is Expressed at All Postnatal Ages but With Lowest Expression at P7 During Hippocampal Development

**FIGURE 2:**
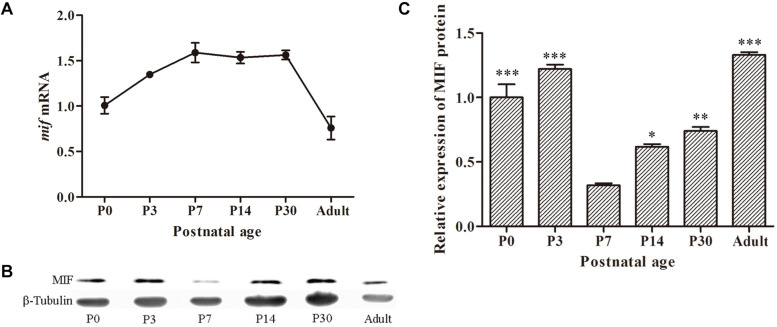
MIF expression in developing hippocampus. **(A)** Relative quantitative real-time PCR for *mif* mRNA extracted from P0, P3, P7, P14, P30, and 3-month hippocampi. Expression level of *mif* mRNA started to rise from P0, reached the peak at P7, and stably expressed until P30 and then declined dramatically to adulthood (3 months). **(B)** Immunoblotting analysis for MIF protein from P0 to 3-month hippocampi. The lowest level of MIF expression was found at P7. β-tubulin was used as a loading control. **(C)** Densitometric analysis from three independent experiments showed that the expression profile of MIF protein during hippocampal development is like an inverted bell with the lowest level at P7 and higher level before or after. Statistical analysis indicated that the expression of MIF at P0, P3, P14, and P30 was significantly increased compared to that at P7 (Tukey multiple-comparisons test). All data were presented as mean ± SEM from three different experiments. ***P < 0.001, **P < 0.01, *P < 0.05.

To investigate the expression profile of MIF during development of hippocampus, we first checked *mif* mRNA by using quantitative real-time PCR at different developmental time points: P0, P3, P7, P14, P30, and 3 months. The results showed that expression level of *mif* mRNA gradually increased from P0 to P7, reached the peak at P7, and stably expressed until P30, and then declined dramatically to adulthood (Figure 2A). Compared with that at P0, *mif* mRNA increased by 0.6-fold at P7 and decreased by 0.4-fold at adult (Figure 2A). Western blotting result demonstrated that MIF was expressed at all postnatal ages (Figure 2B). Densitometric analysis from three independent experiments showed that the expression profile of MIF protein during hippocampal development is like an inverted bell with the lowest level at P7 and higher level before or after (Figure 2C). Compared to that at P7, the MIF expression was increased by 2.8-fold at P3 and 0.9-fold at P14 (Figure 2C). Statistical analysis revealed significant differences between P7 and other time points during development of hippocampus (Figure 2C).

### MIF Is Highly Expressed in Hippocampal Mossy Fibers of P14 Mice

**FIGURE 3:**
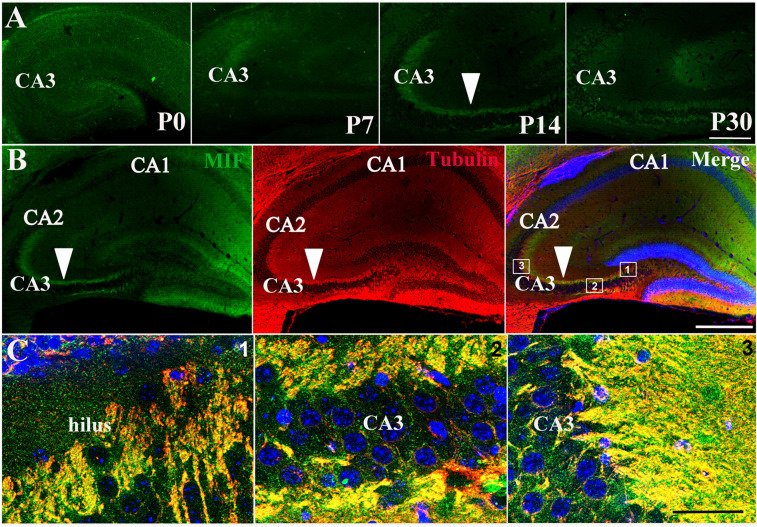
MIF is highly expressed in hippocampal mossy fibers of P14 mice. **(A)** Immunofluorescent detection of MIF (green) in hippocampus at P0, P7, P14, and P30. MIF expression at P7 was the lowest compared to that at P0, P14, and P30 (white arrowhead). **(B)** At P14, there was a strong MIF (green) expression in stratum lucidum of CA3 along pyramidal cell layer. Double-labeling of MIF and tubulin (red) revealed that MIF was perfectly overlapped with tubulin in mossy fibers (white arrowheads). **(C)** High magnifications of merged images from regions indicated in the rectangular boxes in B. Nuclei were counterstained with DAPI (blue). Scale bars in **(A)**: 100 μm, **(B)**: 400 μm, **(C)**: 40 μm.

Immunostaining of MIF in P0, P7, P14, and P30 hippocampus further proved that MIF expression at P7 was the lowest compared to those at P0, P14, and P30 ages (Figure 3A). In addition, we noticed strong MIF expression in stratum lucidum of CA3 from P14 (Figure 3A). To make sure that MIF was located in mossy fiber projections, the axons of granule cells, we performed double-immunolabeling of MIF and tubulin in sections of P14 hippocampus, and results revealed that MIF was perfectly overlapped with tubulin in mossy fibers (Figures 3B,C).

### MIF Interacts With Cytoskeletal Structures

**FIGURE 4:**
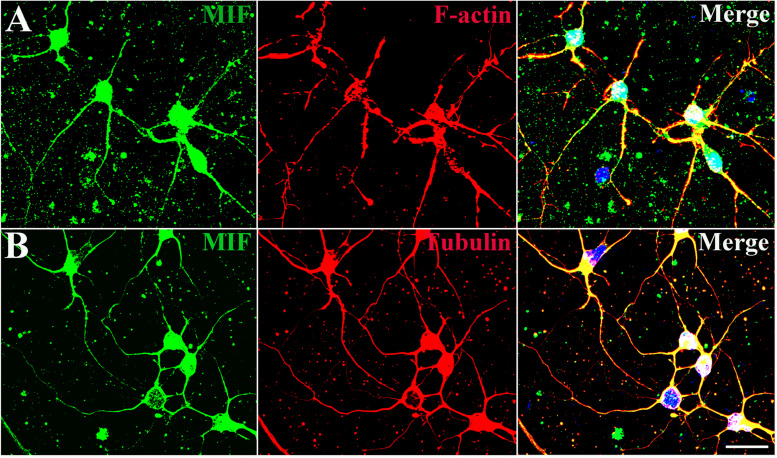
MIF interacts with cytoskeletal molecules in cultured neurons. Hippocampal primary neurons were isolated from P0 and cultured for 5 days *in vitro*. **(A)** MIF (green) was overlapped with F-actin (red). **(B)** MIF (green) was merged with tubulin (red). Nuclei were counterstained with DAPI (blue). Scale bars in **(A,B)**: 20 μm.

In order to explore the relationship between MIF and the nerve fiber structure, immunostaining of MIF and cytoskeletal molecules was performed in cultured neurons. Phalloidin recognizes F-actin (filamentous actin), which forms actin cytoskeleton, and tubulin recognizes microtubule cytoskeleton. Our results clearly showed that MIF colocalized with phalloidin in F-actin and tubulin in microtubules, indicating that MIF interacted with cytoskeletal structures (Figures 4A,B).

### MIF Is Expressed in Both NeuN-Positive and NeuN-Negative Cells in Developing Hippocampus

**FIGURE 5:**
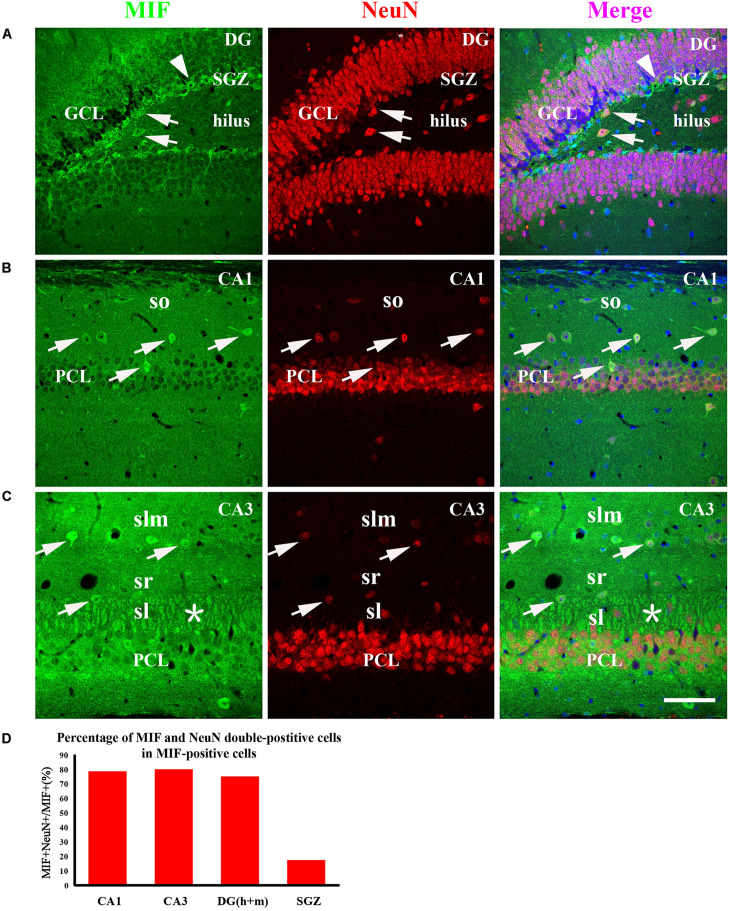
MIF is expressed in both NeuN-positive and NeuN-negative cells in whole hippocampus of 3-month-old mice. MIF-positive cells (green) were scattered in the DG, CA1, and CA3 **(A–C)**. They were neither expressed by NeuN-positive granule cells (red) in the granular cell layer (GCL) in the DG (A) nor by NeuN-positive pyramidal neurons (red) in the pyramidal cell layer (PCL) in CA1 **(B)** and CA3 **(C)**. But they were NeuN-positive cells (**A–C**, white arrows). In DG, except some MIF and NeuN double-labeled neurons scattered in hilus, a densely packed MIF-positive cells (green) with their processes extending into GCL in subgranular zone (SGZ) was observed. However, these cells were NeuN-negative (A, white arrowhead). In CA1 and CA3, MIF and NeuN double-labeled neurons were found sporadically distributed in the stratum oriens (so), stratum lacunosum moleculare (slm), stratum lucidum (sl) (**B,C**, white arrows). In CA3, mossy fibers were positive for MIF in sl, marked by an asterisk **(C)**. Quantitative analysis showed that the percentages of MIF and NeuN double-labeled cells in MIF-positive cells in CA1, CA3, and DG (h + m) were significantly increased compared to that in SGZ **(D)**. Means ± SEM, two-tailed Student’s *t-*test, ***P < 0.001, *n* = 5. DG, dentate gyrus. Scale bars in **(A–C)**: 80 μm.

To examine whether the cells expressing MIF are neurons during development of hippocampus, we first performed double-immunostaining for MIF and NeuN in hippocampal sections from 3-month mice. NeuN is a specific marker for mature neurons. Our results showed that many MIF-positive cells were scattered throughout the whole hippocampus, and a group of MIF-positive cells was accumulated in subgranular zone (SGZ) of dentate gyrus (DG) and formed a densely packed cell layer with long processes extending into GCL (Figure 5A). Those MIF-expressing cells scattered in hilus of DG (Figure 5A) and in stratum oriens (so), stratum radiatum (sr), and stratum lacunosum molecular (slm) of hippocampus proper were found NeuN-positive (Figures 5B,C). However, neither NeuN-positive granule cells in the granule layer (GCL) nor NeuN-positive pyramidal neurons in the pyramidal cell layer (PCL) were seen MIF-positive (Figures 5A–C). Notably, the densely packed cell layer with MIF expression in SGZ showed NeuN-negative (Figure 5A). These results indicated that MIF was expressed in both NeuN-positive and NeuN-negative cells (Figures 5A–C). Here again a strong expression of MIF was observed in mossy fibers in sl attached to the pyramidal neurons in CA3 region (Figure 5C). We took an area of 0.1 mm^2^ in the DG, CA1, and CA3 areas of each hippocampal slice; counted 10 MIF-positive cells; and calculated the percentage of MIF and NeuN double-labeled cells in MIF-positive cells. Five hippocampal slices in total were used. As shown, the percentages of MIF and NeuN double-labeled cells in MIF-positive cells in CA1,CA3 and DG (h + m) were much higher than that of MIF and NeuN double-positive cells in MIF-positive cells in SGZ (Figure 5D).

### MIF Is Expressed by NeuN-Positive GABAergic Interneurons in Developing Hippocampus

**FIGURE 6:**
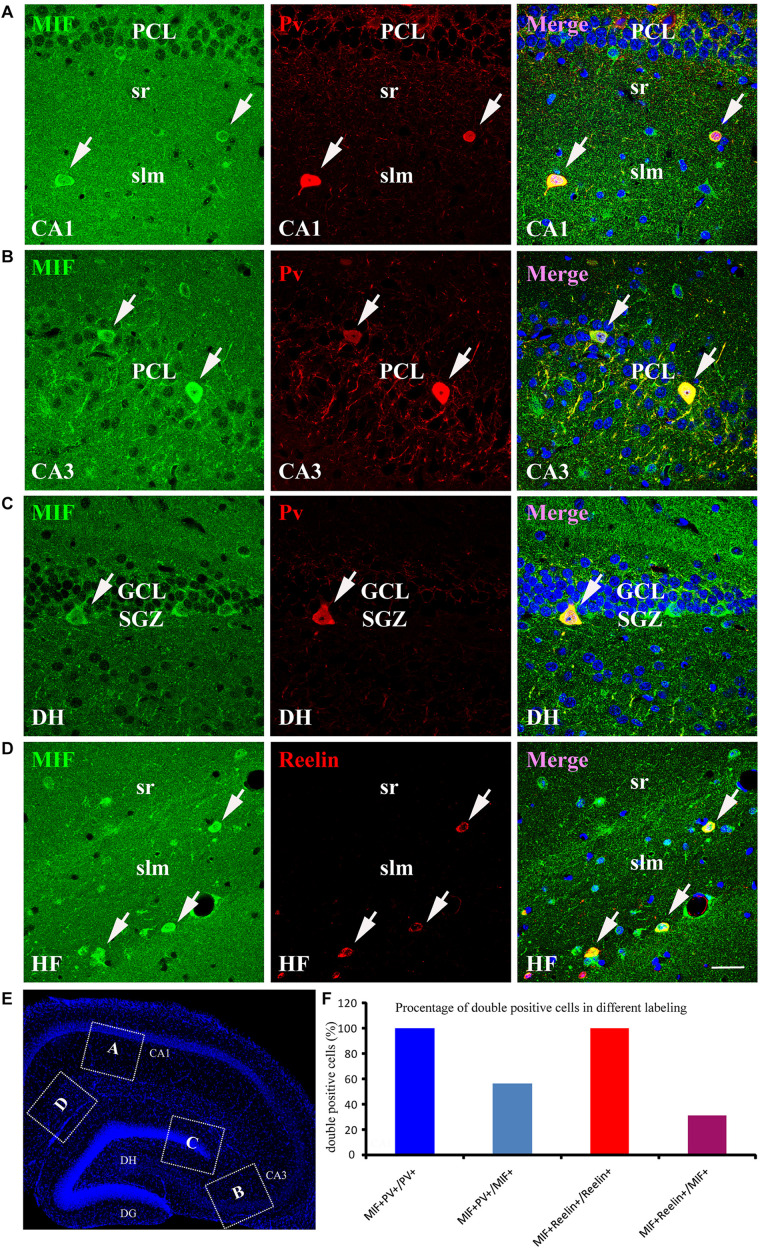
MIF is expressed by NeuN-positive GABAergic interneurons in adult hippocampus. **(A–C)** Double-labeling of MIF and parvalbumin (Pv) in CA1, CA3, and DG of the hippocampus. MIF staining (green) was colocalized with parvalbumin-positive interneurons (red) in stratum radiatum (sr), stratum lacunosum moleculare (slm) of CA1 **(A)**, CA3 pyramidal cell layer (PCL) **(B)**, and DG subgranular zone (SGZ) **(C)**. **(D)** Around the hippocampal fissure (HF), MIF was found located in the Reelin-expressing interneurons (red). These cells were smaller than parvalbumin-positive cells. All double-labeled cells were pointed with white arrows. **(E)** The boxed regions indicated the location of **(A–D)** in the hippocampal slice. Nuclei were counterstained with DAPI (blue). **(F)** Quantification results showed that all parvalbumin or Reelin-positive cells were MIF-positive (MIF^+^Pv^+^/Pv^+^ or MIF^+^Reelin^+^/Reelin^+^), but not all MIF-positive cells were parvalbumin- or Reelin-positive (MIF^+^Pv^+^/MIF^+^ or MIF^+^Reelin^+^/MIF^+^). DG, dentate gyrus; DH, dentate hilus. Scale bars in **(A–D)**: 30 μm.

As the most of MIF and NeuN double-labeled cells in hippocampus were scattered throughout the whole hippocampus, not in the GCL and PCL, and morphologically very similar to interneurons (Figure 5), we speculated that these MIF and NeuN double-positive cells were interneurons. To confirm our supposition, we carried out double-immunolabeling of MIF with parvalbumin (Pv) and Reelin antibodies, respectively, in 3-month-old hippocampus. The results revealed that the majority of MIF-positive cells in hippocampus except the cells in SGZ were colabeled with parvalbumin (Figures 6A–C) or Reelin (Figure 6D). Given that parvalbumin and Reelin are markers for GABAergic interneurons, these results indicated that the scattered MIF-positive cells in hippocampus were GABAergic interneurons. Finally, we counted MIF-positive cells and parvalbumin-positive cells in 0.03 mm^2^ area of CA1 and CA3 region, MIF-positive cells, and Reelin-positive cells in 0.03 mm^2^ area of hippocampal fissure (HF) region. In Figure 6E, the rectangular boxes indicate the regions as shown in Figures 6A–D. Six hippocampal slices in total were used. We found that parvalbumin- or Reelin-positive cells were all MIF-positive, but not all MIF-positive cells were parvalbumin- or Reelin- positive. The percentage of parvalbumin and MIF double-labeled cells in MIF-positive cells was higher than that of Reelin and MIF double-labeled cells in MIF-positive cells (Figure 6F).

### MIF Is Expressed by Neither Immature nor Mature Granule Cells in Developing Hippocampus

**FIGURE 7:**
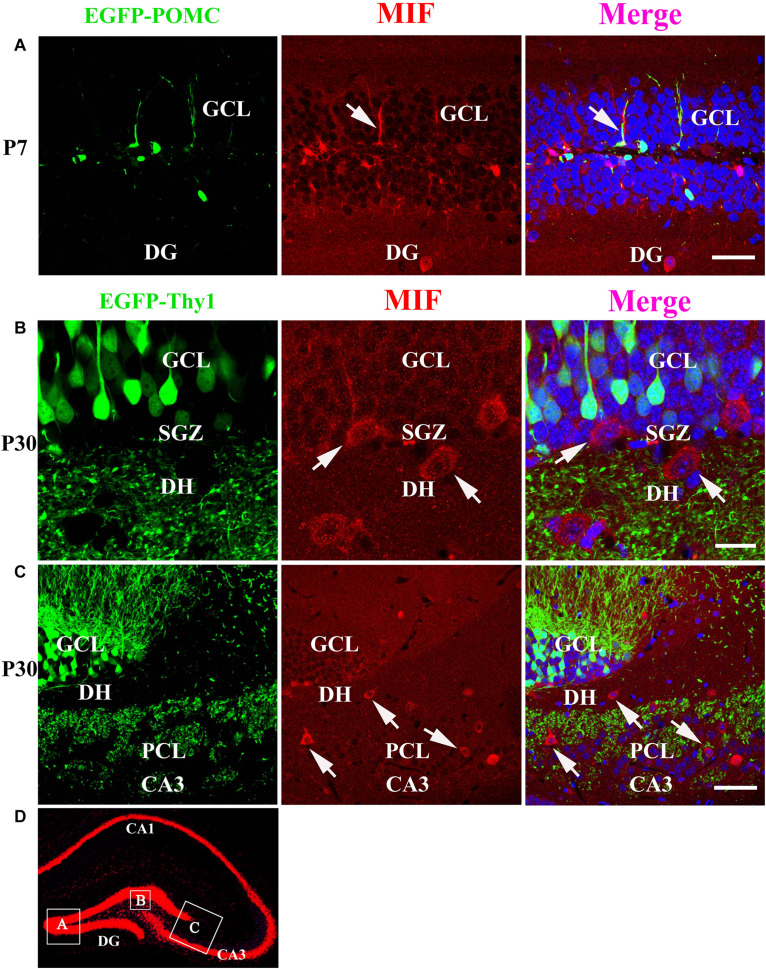
MIF is neither expressed by immature granule cells (P7) nor by mature granule cells (P30) in developing hippocampus. **(A)** P7 POMC-EGFP–positive immature granule cells (green) in GCL were not overlapped with MIF-positive cells (red) distributed in the DG. **(B)** Three-month Thy1-EGFP–positive mature granule cells (green) in 3-month-old mice were not colocalized with MIF-expressing cells (red) scattered in subgranular zone (SGZ) and dentate hilus (DH). MIF-positive cells in SGZ and DH displayed giant morphology prompting them as interneurons. **(C)** In CA3 of 3-month Thy1-EGFP hippocampus, many MIF-positive cells (red) were distributed in stratum lucidum (sl) and pyramidal cell layer (PCL). No overlapping of EGFP (green) and MIF (red) was observed. Some of MIF-positive cells were indicated with white arrows in **(A–C)**. **(D)** The rectangular boxes in hippocampus overview represent the regions shown in **(A–C)**. DG, dentate gyrus. Scale bars in **(A)**: 50 μm; **(B)**: 20 μm: **(C)**: 50 μm.

As MIF was strongly expressed in mossy fibers (Figures 3B,C) and previous study has suggested that NeuroD-positive immature granule cells express MIF (Conboy et al., 2011), wherefore, to find out whether the granule cells express MIF, we used P7 POMC-EGFP and 3-month-old Thy1-EGFP transgenic mice, in which most immature and mature granule cells are labeled by EGFP, respectively. In the transgenic mice, POMC promoter-driven EGFP expression in hippocampal granule cells remains only 1 week after generation. One week after, granule cells began to express EGFP under Thy1 promoter. Therefore, they can represent immature granule cells and mature granule cells. MIF immunostaining showed that neither POMC-EGFP–labeled immature granule cells nor Thy1-EGFP–positive mature granule cells express MIF (Figure 7). POMC-EGFP–positive immature granule cells in GCL were not overlapped with any MIF-positive cells distributed in DG (Figure 7A). Thy1-EGFP–positive mature granule cells in GCL were also MIF-negative (Figures 7B,C). Moreover, MIF-positive cells scattered in SGZ and hilus of DG (DH) displayed giant morphology prompting them as interneurons (Figures 7B,C). In Figure 7D, the rectangular boxes indicate the regions shown in Figures 7A–C.

### GFAP- and BLBP-Positive Neural Precursor Cells in SGZ Express MIF

**FIGURE 8:**
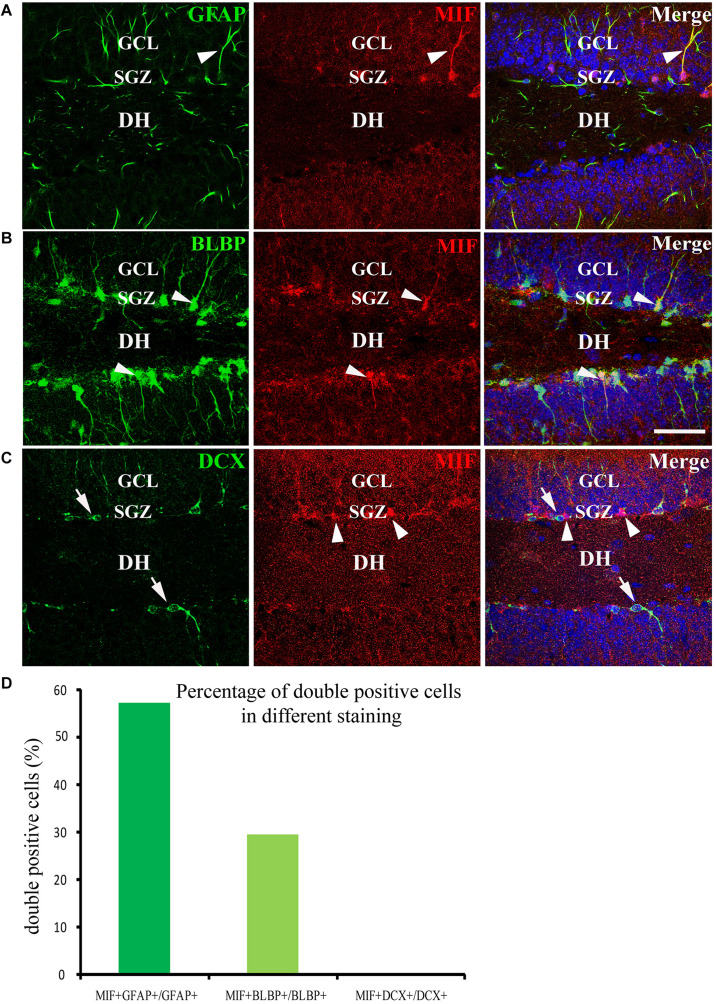
MIF is maintained in neural precursor cells. **(A–C)** Double-labeling of MIF with GFAP, BLBP, and DCX, respectively, indicated that MIF (red) was maintained in fibers (green) of GFAP- positive radial glial cells **(A)** and BLBP-positive neural precursor cells (green) located in the subgranular zone (SGZ) of the DG (**B**, white arrowheads). MIF (red, white arrowheads) was not observed in DCX-positive newly generated neurons (green, white arrows) situated in SGZ of the DG **(C)**. **(D)** Quantification results showed the percentage of GFAP and MIF double-labeled cells in GFAP-positive cells, BLBP and MIF double-positive cells in BLBP-expressing cells, and DCX and MIF double-labeled cells in DCX-positive cells. DH, dentate hilus. Scale bars in **(A–C)**: 40 μm.

We have mentioned above that many MIF-positive cells accumulated in SGZ of DG and formed an MIF-positive cell layer. Interestingly, in contrast to MIF-positive cells in other regions, the MIF-positive cells in SGZ were NeuN-negative and gave rise to MIF-positive processes pointing to molecular layer of DG (Figure 5A). It is well known that SGZ is the place where the neural stem cells and newly generated neurons are located, and neurogenesis occurs for whole life (Zhao et al., 2007). Thus, we wondered whether the neural stem cells or newly generated neurons express MIF. To test these possibilities, we performed double-labeling of MIF with GFAP, BLBP, and DCX, respectively, in 3-month-old hippocampus. Our results showed that most of MIF-positive cells in SGZ were colabeled by GFAP (Figure 8A) and BLBP (Figure 8B), markers for neural stem cells. We also found some MIF-positive astrocytes around the small blood vessels in the hippocampus proper (data not shown). In contrast, no MIF-positive cells in SGZ were found to be colabeled with DCX (Figure 8C), a marker for newly generated immature neurons. It is well known that GFAP is expressed by radial glial cells (neural stem cells), which are located in SGZ of the DG and act as neural precursor cells in developing hippocampus. In adulthood, most radial glial cells are transformed into astrocytes, whereas GFAP is retained by astrocytes. However, there are still some GFAP-positive radial glial cells in the SGZ acting as neural precursor cells (Zhao et al., 2004; Brunne et al., 2010). BLBP is another marker of neural precursor cells, which recognizes late differentiated radial glial cells (Hartfuss et al., 2003; Brunne et al., 2010). DCX is a microtubule-associated protein and expressed by newly generated neurons in SGZ of DG (Zhao et al., 2007). Our findings suggested that MIF is involved in maintenance of the feature of neural stem cells and cell proliferation. To quantify our results, we counted the number of GFAP-positive cells, BLBP-positive cells, and DCX-positive cells in 0.03 mm^2^ area in DG respectively. Six hippocampal slices were counted. We found that the percentage of GFAP and MIF double-labeled cells in GFAP-positive cells was higher than that of BLBP and MIF double-positive cells in BLBP-expressing cells; no double-labeled cells with DCX and MIF were found in DG (Figure 8D). These results further indicated that MIF is maintained in GFAP- or BLBP-positive neural stem cells during hippocampal development.

### MIF Antagonist Suppresses Cell Proliferation in the Developing Hippocampus

**FIGURE 9:**
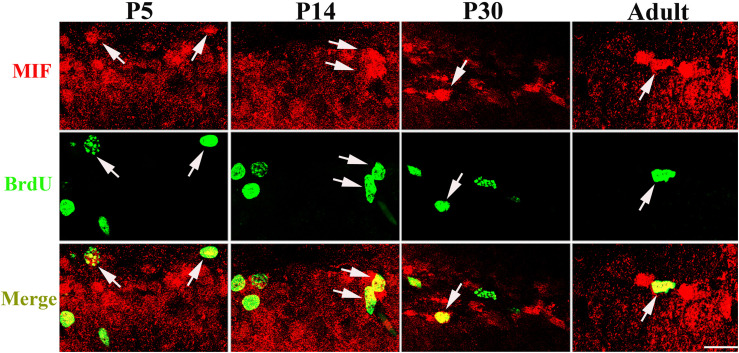
MIF is involved in cell proliferation in developing hippocampus. Injection of BrdU into P5, P14, P30, and 3-month-old mice revealed that MIF (red) appeared in some BrdU-positive (green) newborn cells in subgranular zone of the DG (white arrows). Scale bar: 20 μm.

**FIGURE 10:**
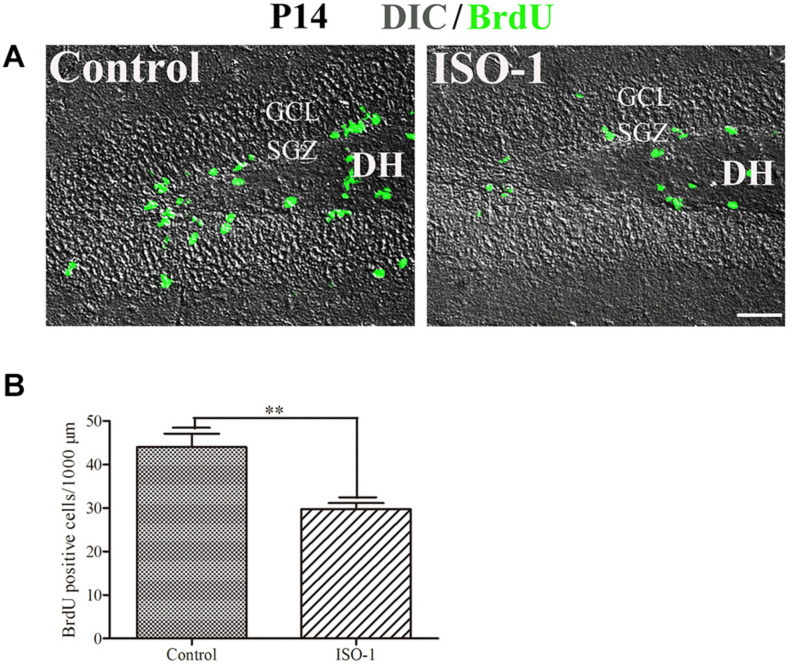
Blocking of MIF impaired cell proliferation in P14 hippocampus. **(A)** Differential interference contrast microscope imaging with BrdU fluorescent imaging showed that MIF antagonist ISO-1 reduced BrdU-positive cells (green) in SGZ of the DG from P14 mice compared to that in control mice. **(B)** Statistical analysis exhibited significant difference of BrdU-positive cells in ISO-1–treated and control hippocampus. Data were presented in bar graphs showing means ± SEM, **P < 0.01, *n* = 12. DH, dentate hilus. Scale bar in **(A)**: 50 μm.

Our data suggested that MIF is involved in cell proliferation. Conboy et al. (2011) have also shown that lack of MIF causes impairment of cell proliferation of adult hippocampus. So we were wondering whether MIF controls cell proliferation of neural stem cells in SGZ of DG during hippocampal development. Then we injected BrdU into P5, P14, P30, and 3-month-old adult mice 2 h before the animals were sacrificed and conducted double-labeling of MIF and BrdU in hippocampus. As revealed in Figure 9, BrdU emerged in MIF staining partially in SGZ of DG at all stages. By injection of ISO-1, an antagonist of MIF, into P5 mice for 9 consecutive days, we found a remarkable reduction of BrdU-positive cells in SGZ of DG in treated mice compared to that in control mice (Figure 10A). Statistical analysis was shown in Figure 10B. Our results indicate that inhibition of MIF impairs hippocampal cell proliferation at an early stage.

### MIF Antagonist Hinders Neurite Outgrowth *in vitro*

**FIGURE 11:**
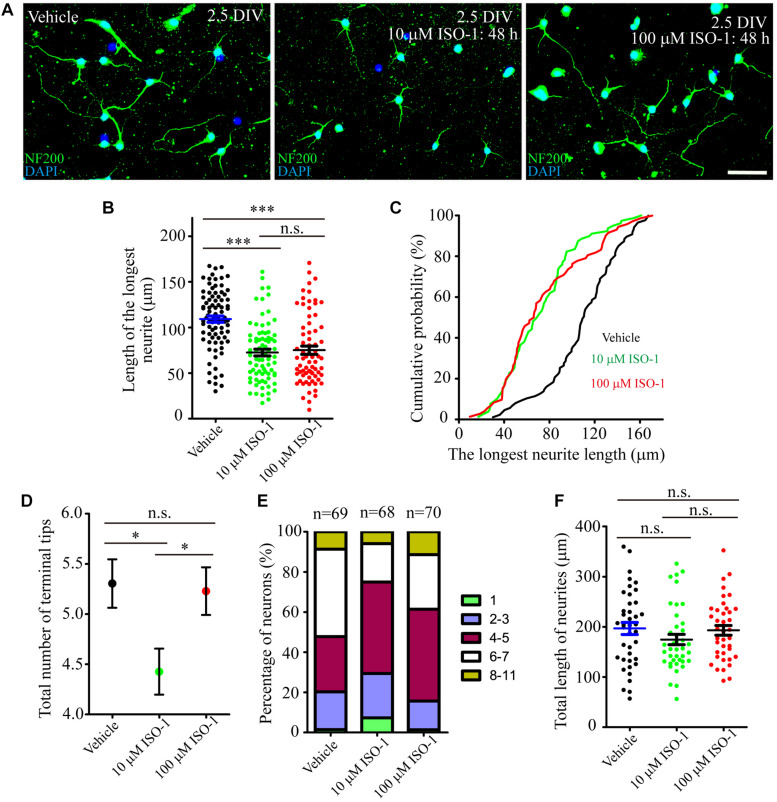
MIF antagonist hinders neurite outgrowth *in vitro*. **(A)** Fluorescent microscopic imaging of cultured neurons labeled with neurofilament 200 revealing that 10 μM ISO-1 affected neurite outgrowth. **(B,C)** Statistical analysis and cumulative probability results demonstrated that both 10 and 100 μM ISO-1 strongly decreased the length of the longest neurites of cultured neurons, and the difference was significant. **(D)** 10 μM ISO-1 treatment significantly reduced the number of terminal tips of neurites. **(E)** The percentage of neurons with different number of neurites in control and ISO-1–treated cultures was evaluated. **(F)** No significant difference of total length of neurites between control and ISO-1–treated neurons. Statistical analysis was performed by Kruskal–Wallis with Dunn post test in **(B)** (n.s., no significance; ***P < 0.001); one-way analysis of variance with Tukey post test in **(D)** (n.s., no significance; *P < 0.05) and **(F)** (n.s., no significance). Scale bar in **(A)**: 50 μm.

During hippocampal development, MIF was expressed in mossy fibers projected from granule cells to pyramidal neurons in CA3 region suggesting that MIF might participate in the regulation of axonal outgrowth (Figure 3). To test this possibility, we treated primary neuronal cultures with ISO-1 using different concentrations (10 and 100 μM) for 2 days. After fixation with 4%PFA in 0.1 M PB, cultures were visualized with neuronal marker neurofilament 200 and photographed. Three independent fields on a coverslip were imaged. At least three coverslips for each experiment were analyzed. Compared with control condition, treatment at both concentrations of ISO-1 seemed to affect neurite outgrowth (Figure 11A). To verify the extent of the effect, we measured the length of processes. The statistical analysis and cumulative probability results showed that 10 and 100 μM ISO-1 strongly decreased the length of the longest neurites of cultured neurons (Figures 11B,C). To identify the number of total neurites, we counted the terminal tips of neurites. The result revealed that 10 μM ISO-1 treatment led to a reduction in number of neurites, with significant difference compared with that in control and 100 μM ISO-1–treated neurons (Figure 11D). These data implied that appropriate concentration of ISO-1 is critical to block MIF function. In addition, the percentage of neurons with different number of neurites in control and ISO-1–treated cultures was evaluated, and the total length of processes except the longest neurite was measured (Figure 11E). No significant difference between control and ISO-1–treated neurons was observed (Figure 11F). It is well known that the longest protrusion from a neuron is axon, and the other protrusions differentiate to dendrites. Therefore, our results indicate that MIF might participate in the regulation of mossy fiber projection and dendritic bifurcation. Blocking of MIF with ISO-1 inhibited axonal elongation and dendritic formation.

## Discussion

MIF has been found to be expressed in the neocortex, hippocampus, hypothalamus, cerebellum, choroid plexus, and spinal cord in the CNS (Nishibori et al., 1997; Bacher et al., 1998; Ogata et al., 1998; Vedder et al., 2000; Zhang et al., 2019) and up-regulated in AD and Parkinson disease (Nicoletti et al., 2011; Zhang et al., 2019). Hippocampus is responsible for learning and memory and involved in lots of neurological diseases. However, the spatiotemporal expression pattern of MIF in developing hippocampus has remained unknown. Herein, we examined the expression levels of MIF during hippocampus development both in mRNA and protein quantitatively. We found that MIF is expressed throughout the development of the hippocampus during the postnatal period. Among the selected time points, the lowest level of MIF protein was detected at P7, whereas the higher level of MIF was found both at earlier and later stages. However, our quantitative real-time PCR revealed that the peak of *mif* mRNA was between P7 and P30. These results suggest that there was a mismatch between the levels of MIF protein and *mif* transcript in hippocampus, which was similar with the expression pattern of MIF in the neocortex (Zhang et al., 2014). We speculate that during development, neuronal migration requires a lot of MIF. One week after birth, newly generated neurons finish their migration, and the MIF requirement is reduced. After the neurons grow dendrites and axons and form synaptic connections with their target cells, the outgrowth of neurites results in increased requirement of MIF. For the stably expressed *mif* mRNA during P7 until P30, our explanation is that from P7 on a vast amount of MIF is used for synaptogenesis, and *mif* mRNA is ready for producing MIF proteins in case needed. It has reported that MIF promotes cancer cell migration in human lung adenocarcinoma and esophageal squamous cell carcinoma (Rendon et al., 2007; Liu et al., 2018). Therefore, it is necessary for the future study to investigate the role of MIF on cytoskeletal dynamics in migrating neurons.

GFAP is a marker for radial glial cells located in the ventricular zone of the cerebral cortex and in the SGZ of the DG during development. They extend their radial fibers as scaffold for the migrating neurons. After the migration, the majority of radial glial cells transform into astrocytes (Zhao et al., 2004; Brunne et al., 2010). In our present study, MIF was found in GFAP-positive and BLBP-positive radial glial cells, which act as neural precursor cells located in SGZ of DG, but not in DCX-positive newly generated neurons in SGZ, suggesting that MIF plays a role in maintenance of neural stem cells and might be involved in cell proliferation. Colocalization of MIF and GFAP in astrocytes has been proved by other researchers (Ogata et al., 1998; Conboy et al., 2011; Li et al., 2015). BrdU has long been used as a sign of cell division and a marker to investigate the neurogenesis and gliogenesis in adult hippocampus (Zhao et al., 2007; Conboy et al., 2011). We found that 2 h after injection BrdU has already been detected in some MIF-positive cells in hippocampus prepared from P5 to 3-month-old mice, and blocking of MIF function in P14 mice reduced the number of BrdU-expressing cells in hippocampus. This result further confirmed the involvement of MIF in cell proliferation during development (Conboy et al., 2011). As MIF was found in GFAP-positive and BLBP-positive neural stem cells and astrocytes around brain blood vessels, not in DCX-positive newly generated neurons, we speculated that MIF maintains the feature of neural stem cells and participates in cell proliferation of neural stem cells during hippocampal development.

To find out whether MIF is expressed in the somata of granular cells in hippocampus during development, we used POMC-EGFP and Thy1-EGFP transgenic mice. Our results revealed that neither POMC-EGFP–labeled immature granule cells nor Thy1-EGFP–labeled mature granule cells in DG express MIF. These results are controversial with the previous study showing that only NeuroD-positive immature cells are MIF-positive in DG (Conboy et al., 2011). We conjectured that either we missed the earlier moment at which NeuroD and MIF proteins are expressed equally in cytoplasm by granule cells in DG, or MIF protein is quickly transported to the terminal of the mossy fiber after production, resulting in a very weak signal of MIF in the cytoplasm of MIF, making it difficult to detect (Nishibori et al., 1996; Ogata et al., 1998). It is worth noting that, in our experiments, MIF immunoreactive labeling in mossy fibers was very strong. In addition, the dual-labeling of MIF and NeuN (markers of mature neurons) further proved that MIF is unable to be detected in somata of granule cells and pyramidal neurons of the hippocampus during development. However, we did find that some NeuN-positive cells scattered in whole hippocampus and strongly expressed MIF in their somata. The morphological features of these cells and the immunolabeling for parvalbumin and Reelin evidenced that they were GABAergic interneurons. And it has been reported that MIF is also expressed by GABAergic interneurons in the cerebral cortex (Inácio et al., 2011; Zhang et al., 2014). GABAergic interneurons play a profound role in regulating cell excitability and interact with glutamatergic pyramidal cell inputs in a domain-specific manner and support synaptic temporal dynamics (Bennett and Zukin, 2004; Klausberger and Somogyi, 2008). This makes us more believe that MIF expressed at mossy fibers buttons may regulate the synapse dynamics of pyramidal neurons in CA3. Interestingly, our previous study has shown that granule cell dispersion in temporal lobe epilepsy patients is due to reduced Reelin expression by GABAergic interneurons (Chai et al., 2014). Studies from other laboratories have demonstrated that parvalbumin-positive interneurons prevent stress-induced synapse loss, and MIF is decreased when parvalbumin is increased by enriched environment in the brain following experimental stroke (Inácio et al., 2011; Chen et al., 2018). Taken together, it is likely that Reelin and parvalbumin stabilize synaptic structure, whereas MIF promotes cytoskeletal dynamics (Chai et al., 2009, 2014, 2016). Considering that the expression intensity of MIF in principal neurons and interneurons is different, we speculate that GABAergic interneurons may express more MIF than principal neurons, or they have different ways of secreting MIF and different mechanisms of action of MIF on target cells.

Mossy fibers are axons of dentate granule cells projecting to the CA3 region. In the present study, we confirmed that MIF was expressed in the mossy fibers at early postnatal stages. Thus, it raises the question whether MIF affects axonal outgrowth. To verify this possibility, cultured neurons were treated with MIF antagonist ISO-1. Results showed that suppression of MIF with 10 μM ISO-1 hindered extension and arborization of neurites. We conclude that a suitable ISO-1 concentration is essential for the function of MIF. Coincidentally, treatment with MIF antibody delays nerve regeneration after sciatic nerve injury (Nishio et al., 2002). It is interesting enough that MIF has been reported to direct initial neurite outgrowth from the statoacoustic ganglion to the developing inner ear, and recombinant MIF promotes directional neurite outgrowth of chick and mouse statoacoustic ganglion (Bank et al., 2012). This discovery strengthens our future exploration of the role and mechanism of MIF in regulating nerve fiber dynamics.

## Data Availability Statement

The raw data supporting the conclusions of this article will be made available by the authors, without undue reservation, to any qualified researcher.

## Ethics Statement

The animal study was reviewed and approved by the Institutional Animal Care of Hamburg University and Northwest A&F University.

## Author Contributions

WZ, XC, and SZ developed the concept of the study and wrote the manuscript. XC, WZ, and SZ wrote the manuscript. All authors contributed to the article and approved the submitted version.

## Conflict of Interest

The authors declare that the research was conducted in the absence of any commercial or financial relationships that could be construed as a potential conflict of interest.
